# Identifying Chinese social media users' need for affect from their online behaviors

**DOI:** 10.3389/fpubh.2022.1045279

**Published:** 2023-01-10

**Authors:** Hong Deng, Nan Zhao, Yilin Wang

**Affiliations:** ^1^Institute of Psychology, Chinese Academy of Sciences, Beijing, China; ^2^Department of Psychology, University of Chinese Academy of Sciences, Beijing, China

**Keywords:** need for affect, social media, online behavior, mental health, machine learning, Extreme Gradient Boosting

## Abstract

The need for affect (NFA), which refers to the motivation to approach or avoid emotion-inducing situations, is a valuable indicator of mental health monitoring and intervention, as well as many other applications. Traditionally, NFA has been measured using self-reports, which is not applicable in today's online scenarios due to its shortcomings in fast, large-scale assessments. This study proposed an automatic and non-invasive method for recognizing NFA based on social media behavioral data. The NFA questionnaire scores of 934 participants and their social media data were acquired. Then we run machine learning algorithms to train predictive models, which can be used to automatically identify NFA degrees of online users. The results showed that Extreme Gradient Boosting (XGB) performed best among several algorithms. The Pearson correlation coefficients between predicted scores and NFA questionnaire scores achieved 0.25 (NFA avoidance), 0.31 (NFA approach) and 0.34 (NFA total), and the split-half reliabilities were 0.66–0.70. Our research demonstrated that adolescents' NFA can be identified based on their social media behaviors, and opened a novel way of non-intrusively perceiving users' NFA which can be used for mental health monitoring and other situations that require large-scale NFA measurements.

## 1. Introduction

The need for affect (NFA) is defined as individual differences in motivation to approach or avoid emotional stimulation, which consists of two related but distinct aspects: a motivation to experience emotionality (NFA approach) and a motivation to avoid experiencing emotionality (NFA avoidance) (1). Similar to personality traits, it is regarded as a relatively stable intrinsic character of human nature (1, 2). NFA could help to explain individuals' differences in many critical mental aspects such as mental health (3–9), information preferences and decision making (10–14), social attitude [e.g., evaluation of self and others (15–18), attitudes toward brands (19), attitudes toward job (20), attitudes toward the country (21), drugs (22)] and so on. It has also been found that NFA influences individual reactions to media and entertainment (23, 24), political beliefs and ideology (16, 25), legal decisions (26) and risk taking capacity (27).

The existing research has found that NFA has a significant correlation with negative emotionality and some mental health symptoms (1, 2, 6, 26). For example, the preference to avoid emotions has been demonstrated to be positively associated with alexithymia, negative affect and affective instability (1). It has been shown that NFA avoidance is positively associated with depression, anxiety, stress, lower levels of wellbeing, posttraumatic stress symptoms, psychological exhaustion and indolence (6), indicating that NFA avoidance is a risk factor for poor mental health and burnout.

Specific to suicide behavior and suicide proneness, both NFA approach and NFA avoidance are associated with a higher risk of suicide in nonclinical samples (3–5, 7–9). In addition, NFA plays a mediating role in mental health symptoms and the risk of suicide (5, 7, 8). Specifically, NFA avoidance has been demonstrated to have amplifying effects on other risk factors of suicide such as depression (28), whilst NFA approach could serve as a protective factor in the depression-suicide attempt link (9) and potentially facilitate positive subjective wellbeing (6). NFA could also affect the training effect of suicide prevention (8). Hence, NFA scores could be used to estimate the suicide risk and even act as an early-warning sign for mental health care. It could also be an influential factor when developing novel interventions.

Previous studies have also demonstrated that NFA could influence individuals' preferences for information selection and processing, thus affecting the formation and transformation of a person's attitude (1, 12). Individuals high in NFA tend to rely upon emotional information in attitude formation and the regulation of behavior (1, 12–14). Emotion approach and emotion avoidance have significant and differential effects on the conversion of emotion experiences into attitudes (13). Therefore, NFA may have a significant influence on the application of internet-based psychological and health interventions. By matching the application's information expression form and human-computer interaction mode to an individual's NFA, it will be easier to achieve better results in developing healthy attitudes and behaviors.

As a means of measuring NFA, a 26-item Need for Affect Questionnaire was developed by Maio and Esses (1) and proved to have good reliability and validity. This scale is widely used in studies and applications (16, 20), but it still has some deficiencies, especially in the online scenarios. Firstly, the questionnaire is not suitable for large-scale measurement of online users due to the limitations of participant recruitment and resource consumption. Secondly, no matter how efficiently the questionnaire is implemented and collected, it still takes a long time to get the results for a large-scale survey. Therefore, it is worth exploring and investigating whether and how to use social media behavioral data to identify users' NFA. This study attempted to find a method to recognize users' NFA using their social media data in order to overcome the limitations of self-report methods in social media scenarios.

Today more and more people use social media as their major tool to acquire information and knowledge, display their personal lives and communicate with others. People's psychological traits are becoming more reflected in their online behaviors (29, 30). Recently, researchers started to build machine-learning predictive models to recognize psychological traits based on network behaviors. Several studies have showed that social media users' psychological characteristics are discernible by their online behaviors, such as personality and mental health status. Some typical examples are presented in Table 1. The correlation coefficients between the predicted scores and the real scores in these researches could sometimes achieve medium to high level. This approach has been described as Online Ecological Recognition (OER) (31), and is gradually being validated (32). These results demonstrate the feasibility of recognizing psychological traits based on social media data.

**Table 1:** Examples of the psychological traits prediction studies based on social media data.

**Psychological traits**	**Social media**	**Digital footprints**	**Model**	**Prediction accuracy**	**Citation**
Big-Five personality	Facebook	Language	Regression	0.30–0.46 (*r*)	(63)
	Twitter	Demographics, language	Regression	0.16–0.21 (RMSE)	(64)
	Sina Weibo	Language, behavior	Regression	0.26–0.54 (*r*)	(43, 65, 66)
	Sina Weibo	Language, behavior	Classification	0.70–0.80 (*P*)	(67)
Subjective wellbeing	Sina Weibo	Language, behavior	Regression	0.27–0.60 (*r*)	(44)
Depression	Twitter	Language, behavior	Classification	0.83 (*P*)	(68)
	Facebook	Language	Classification	0.88 (*P*)	(69)
	Sina Weibo	Behavior	Regression	0.61 (MAE)	(70)
Pathological personality	Facebook	Behavior	Regression	0.20 (*r*)	(71)
Anxiety	Sina Weibo	Behavior	Regression	0.54 (MAE)	(70)

Digital footprints, the type of data used in the studies; Prediction accuracy, the prediction accuracy of the model, for regression models, the values are Pearson correlation coefficient between the prediction scores and real scores (*r*), mean absolute error (MAE) or root mean square error (RMSE), for classification models, the values are precision of models (*P*).

Different from emotion and emotion regulation, NFA focuses on people's attitude toward emotion itself (2), and it is a psychological trait related to emotion. Although we do not find any research to date specifically on NFA identification based on network behaviors, many studies have found that users' emotions could be identified based on the blogs and blog comments they posted on social media [e.g., (33, 34)]. In addition, researchers have demonstrated that people with different NFA have different online information preferences. Users with a high level of NFA tend to seek out more affective websites (e.g., emotional pictures and emotional verbiage) than users with a low NFA level (10). Previous studies have also found that the closer the relationship to the sender of the post, the stronger emotional responses would be to the post on social media (35), that is what people with high NFA like to seek (1). To some extent, these results suggest the possibility to identify NFA using social media data.

This study aims to explore the effective way of using social media data to recognize NFA. The study was conducted on Sina Weibo (http://weibo.com/), which is a leading online social network in China. By 2020, nearly 80% of Sina Weibo users were aged under 30 years (36), which means most Weibo users are adolescents and young adults. We studied the feature extraction from Sina Weibo data and the model building based on NFA questionnaire (1) scores for predicting NFA through machine learning. We also tested the reliability and validity of the prediction model by using a method that refers to the reliability and validity test of psychological scales. Our study provides a novel perspective to analyze and measure NFA, and makes up for the shortcomings of questionnaires in specific scenarios.

## 2. Materials and methods

We hypothesized that similar to other psychological traits (e.g., personality), NFA influences individual online linguistic and behavioral patterns. In this study, the correlation between one's NFA and their social media data was analyzed to predict users' NFA based on their social media data. This task was regarded as a regression task in the machine learning area. Based on the regression technique, a machine learning model was developed to predict NFA through features extracted from social media data. Note that the research methods and procedures used were reviewed and approved by the scientific research ethics committee of the Institute of Psychology, Chinese Academy of Sciences. This was done in accordance with ethical specification H15009.

### 2.1. Need for affect questionnaire

The NFA questionnaire is a 26-item psychological scale which was proposed and validated by Maio and Esses (1), and has good validity while conducting on Chinese samples (14, 37). It is composed of two sub scales for NFA approach and NFA avoidance. Each subscale is measured by 13 statements. It has been proved that the NFA questionnaire has reliable and valid psychometric properties and is widely employed in various NFA related studies (1, 16, 20). In this study, we used NFA questionnaire to assess participants' degree of need for affect. It uses a 7-point Likert scale for responses (1 = Strongly disagree to 7 = Strongly agree, with 4 = Uncertain). The scores of NFA approach and NFA avoidance were calculated by adding all the items of each sub scale. After reverse-scoring the 13 items of NFA avoidance, all items were summed for a total NFA score (NFA total) with higher scores indicating a higher NFA degree.

### 2.2. Social media data collection

Nine hundred and ninety-eight active Sina Weibo users were recruited to take part in our experiment from June 2017 to April 2018. Besides the demographic questionnaire which includes information such as gender and age, all the participants completed the NFA questionnaire. They agreed to authorize the researchers to access their public Sina Weibo data after informed consent. Then we downloaded their public Weibo data through the Sina Weibo Application Programming Interface (API) and web crawler. Among the 998 participants, the data of three users were excluded because their accounts were no longer visible during data collection. In total, 995 users' data were successfully obtained, including all contents they posted or reposted, as well as their public information, i.e., profile image, name, location, personal description, friends count, followers count, etc. The 995 users' Weibo data and their NFA questionnaire scores were used as the dataset for the following steps.

### 2.3. Data pre-processing

The collected data has been screened and preprocessed to ensure validity based on the following rules:

1. The users whose total number of posts was less than 100 were dropped out of the study. This was done to ensure each case in the sample had sufficient social media data for data analysis and model building.

2. The amount of time that participants spent answering each question of the questionnaire should be longer than 2 s. If the time was shorter than that, the answer was considered invalid, and the corresponding participant was removed from the study.

3. For one respondent, if all the NFA questionnaire items were rated the same, he/she was dropped from the study.

4. Some of the participants were labeled by Sina Weibo as commercial or VIP users, such as institutional users, fan accounts, advertisers, etc. These participants' Weibo data was also deleted since it generally did not contain personal expression.

**Figure 1:**
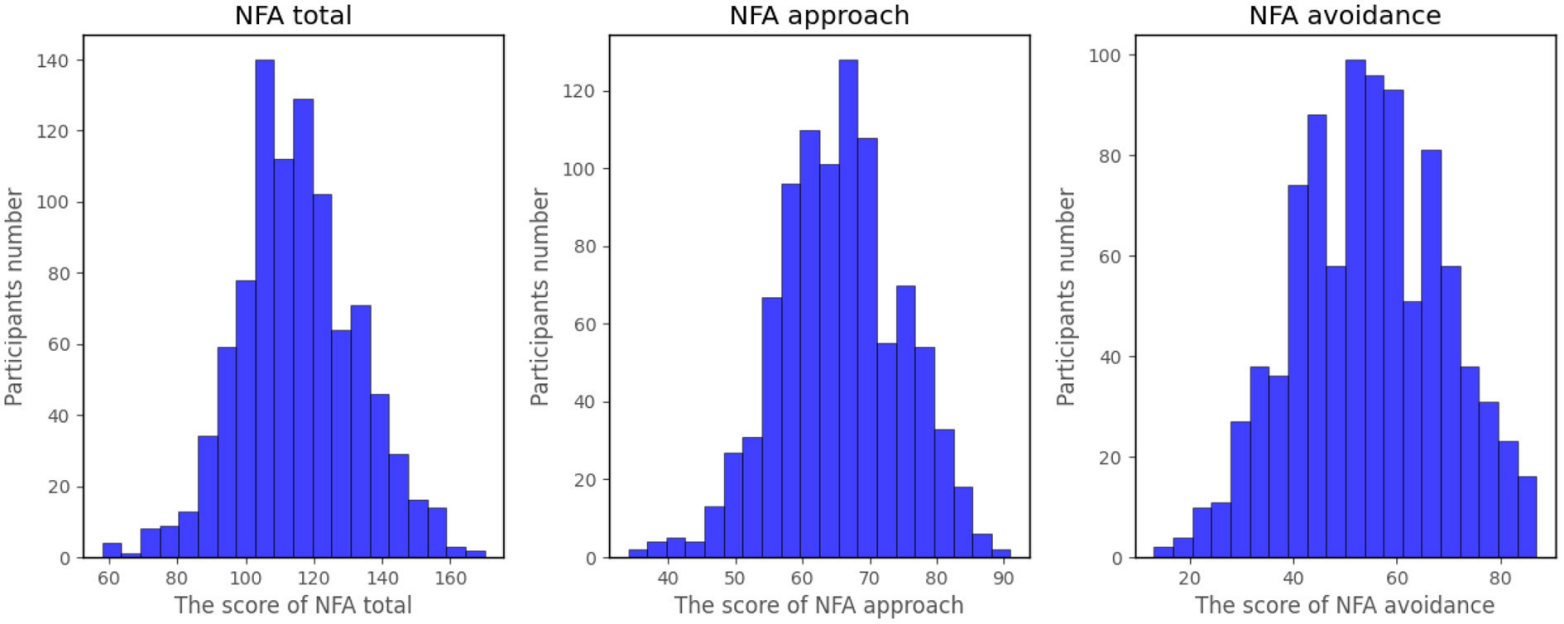
Distribution of NFA scale scores.

After data preprocessing, we finally got 934 valid samples. Their NFA distribution is depicted in Figure 1.

### 2.4. Feature extraction

Based on the assumptions outlined above, namely NFA affects individuals' online behaviors, three types of features were extracted from the collected social media data to build the NFA prediction model.

#### 2.4.1. Demographic features

Previous studies have demonstrated that NFA is correlated with some demographics such as gender and age (1, 2). Therefore, we included gender, age and location in further analysis.

#### 2.4.2. Behavioral features

Behavioral features refer to the characteristics and patterns of social media using behavior. Two types of behavioral features have been extracted as below:

(1) Personal behaviors

Personal behaviors are personalized behaviors during social media use, including: (a) Self-presentation behaviors, referring to the general displaying of personal images and statements online, such as profile names, profile images and brief self-description; (b) Self-expression behaviors, referring to the patterns of posting behaviors such as the average number of microblogs posted/reposted per day, the number of original posts, the ratio of original posts to all posts, etc.; (c) Privacy settings, such as the user's preference on whether or not to receive a private message or comment on blogs from strangers.

(2) Interpersonal behaviors

Interpersonal behaviors refer to the interaction between different users, including the number of friends and followers, bi-following count, the number of @ (being mentioned by others), etc.

#### 2.4.3. Linguistic features

Linguistic features are identified as users' language expression patterns in social media. In this study, each participant's original posts were collected and combined into one text file. After segmenting textual data, word frequencies were calculated as linguistic features. Each word frequency was based on a category outlined in psycholinguistic lexicons. In this study, two psycholinguistic lexicons were used to extract linguistic features.

(1) SCLIWC features

Linguistic Inquiry and Word Count (LIWC) (38) is a text analysis program based on psychologically meaningful categories which has been widely used in existing research to extract linguistic features from social media data like Twitter and Facebook (32, 39). It has been proved to be an effective method to analyze psychological semantics through text (40). The simplified Chinese version LIWC (SCLIWC) (41, 42) was adopted in this study, which has been proved valid to analyze the linguistic features on Sina Weibo, and used in some studies predicting psychological traits such as personality and mental health status (43, 44). It consists of 91 psychologically meaningful word categories in total, such as “personal pronouns,” “quantifiers,” “social processes,” “affective processes,” “cognitive processes,” “achievement,” “home,” “assent,” etc. Therefore, in our study, 91 SCLIWC features were extracted for each participant.

(2) Weibo-5BML features

Considering that NFA is an emotion-related psychological trait, we also used Weibo Basic Mood Lexicon (Weibo-5BML) which has been designed for emotion analysis of Weibo data (45). There are five word categories in this lexicon: “happiness,” “sadness,” “fear,” “anger,” and “disgust.” And 5 Weibo-5BML features were obtained per participant.

In total, 122 features were extracted for each sample, including 3 demographic behaviors, 23 behavioral features and 96 linguistic features.

### 2.5. Model training

#### 2.5.1. Data re-sampling

As shown in Figure 1, the scores of NFA total, NFA approach and NFA avoidance are all approximately normal distributions. We divided the whole data into three groups. The low or high group was defined as 1 SD (standard deviation) below or above the mean, the remaining data was categorized into the intermediate group. There has primarily been research on high and low NFA groups since high or low NFA is often related to mental and behavioral consequences (26), which is the goal of model prediction. However, unevenly distributed data may cause algorithms biased toward the majority group (46–49) —which is the intermediate group in our study. For this reason, re-sampling is necessary to obtain a more balanced sample, and in our study random under-sampling was employed prior to model training. Approximately a similar number of random subsets as the other two groups were selected from the intermediate group. These subsets were then joined with the low and high groups to form the final training data set.

#### 2.5.2. Modeling process

After data re-sampling, eight machine learning algorithms were used to build prediction models based on the selected features, i.e., Linear Regression (LR), Support Vector Regression (SVR), Gradient Boosting Regression (GBR), Random Forest Regression (RF), Least absolute shrinkage and selection operator (LASSO), Ridge Regression (Ridge), Extra Trees Regression (ETR) and Extreme Gradient Boosting (XGB). During model training, five-fold cross validation was conducted to maximize the utilization of the dataset and avoid overfitting, and the grid search method was used to tune the model parameters.

**Figure 2:**
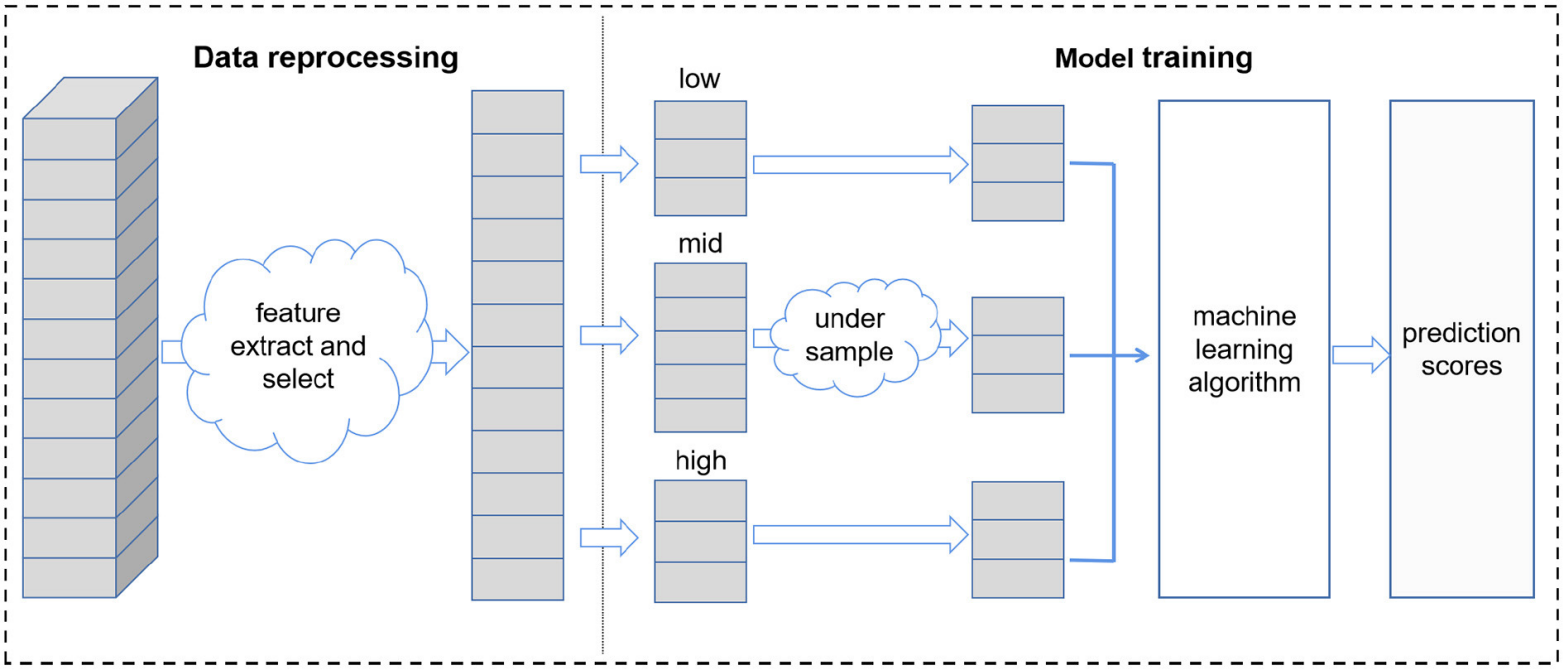
The process of model training.

The whole model training process is presented in Figure 2.

### 2.6. Feature selection

There were 934 samples collected and each sample had 122 features based on feature extraction. Inputting all of these features into the model would make it more complex, but less generalizable due to the fact that not all features were equally useful. In order to maximize the performance of the model and avoid model overfitting, we first selected the features which contributed the most to the labels represented by NFA questionnaire scores in our study. The Random Forest (RF) algorithm (50) is an improvement in Bagging algorithm with the multilevel decision tree, which is often used for feature selection in developing the data-driven model (51–53). In this study, the final feature subset used in model training was selected through the following two steps:

1. If a feature shared the same value across most participants and was not useful in distinguishing them, it would be filtered out to reduce feature space.

2. All the features which were screened out in the first step were ranked through the RF algorithm. The tree-based strategies used by RF algorithm naturally rank how important the feature is during model building (50, 52). The importance of a feature was measured by the score for the feature output by RF. Features with the lowest score were eliminated.

After the above two steps, the number of features selected for each dimension was: 19 for NFA total, 19 for NFA approach and 15 for NFA avoidance.

### 2.7. Model evaluation

As a new method to measure NFA, the performance of the prediction models should be tested. Except for existing model evaluation methods, such as R-square/R^2^ (the proportion of the variability in the response variable), the reliability and validity of the predictive models were also evaluated by an improved psychometric method which is suitable for machine-learning models (54).

1. Criterion validity

With a trained model, the selected social media behavioral features could be used as the input, and the output of the model was the predicted NFA scores. The questionnaire scores of each NFA dimension were used as the validity criterion. The criterion validity of the model was assessed by the Pearson correlation coefficients between predicted scores and the scores of the NFA questionnaire.

2. Split-half reliability

The data set was randomly divided into two subsets. The one containing 655 cases (70% of the sample) was used as the training set to build and save a test model, and the other with 279 cases (30% of the sample) was used as the test set. For each sample in the test set, the participant's posts were sorted by the time they posted and separately merged those with odd numbers as “odd-data” and those with even numbers as “even-data.” Then the odd-data and even-data were used as the inputs of the test model separately to get two outputs as “split-half” scores. The split-half reliability for each model was assessed with the Pearson correlation coefficient between the “split-half” scores.

## 3. Results

### 3.1. Demographic information

After data screening and preprocessing, a total of 934 participants (female = 731) were involved in the study. They ranged in age from 15 to 73 (M = 23, SD = 4.03). The demographic information of these individuals is shown in Table 2.

**Table 2:** Demographic information of participants.

**Demographic information**	**n**	**%**
**Gender**		
Female	731	78.27
Male	203	21.73
**Age**		
~25	767	82.12
25–30	138	14.78
31–40	22	2.35
41~	7	0.75
**Location**		
Domestic	834	89.29
Oversea	61	6.53
Missing data	39	4.18
**Educational background**		
Middle school	7	0.75
High school	64	6.85
Associate or bachelor	598	64.03
Master or above	265	28.37
**Total**	934	100

### 3.2. Questionnaire scores of NFA

In general, participants possessed higher scores on NFA approach scale (M = 65.42; SD = 9.22; Range = 34–91) compared to the scores on NFA avoidance scale (M = 54.44; SD = 14.39; Range = 13–87). The two dimensions were not correlated in our data (*r* = −0.056, *p* = 0.09). As shown in Table 3, there was no significant gender difference in NFA approach, avoidance and total scores.

**Table 3:** Distribution of NFA Questionnaire scores.

	**Female**		**Male**					

	**M**	**SD**	**M**	**SD**	***t***	**df**	**p**	**95%CI**
NFA total	115.38	17.62	113.51	17.15	1.345	932	0.94	[−0.858, 4.597]
NFA approach	65.76	9.34	64.2	8.69	2.134	932	0.21	[0.125, 2.991]
NFA avoidance	54.37	14.52	54.68	13.95	−0.272	932	0.84	[−2.553, 1.931]

M, mean; SD, standard deviation.

### 3.3. Selected features

Different features were selected for different dimensions after feature selection. We classified selected features according to different feature types and listed them in Table 4. We also performed correlation analysis on these features by calculating the Pearson correlation coefficients between the features and the NFA questionnaire scores. The results indicate that some of these features are significantly correlated with the questionnaire scores (see Table 4).

**Table 4:** Selected features for modeling.

	**L**				

	**SCLIWC**	**Weibo-5BML**	**D**	**B**	**Total**
NFA total	Qmark (*r* = −0.11)***, money, dash (*r* = −0.07)*, death (*r* = −0.08)**, apostrophe, ingest, inclusive, religion, achieve, we, certain, sexual, love, multiFun	NA	NA	Self-description length, bi-followers/friends (*r* = −0.07)*, general emoticons-total words ratio(*r* = 0.10)***, posts posted at night/total posts, posts contain URL/total posts(*r* = 0.08)*	19
NFA approach	PPron (*r* = 0.12)***, Qmark (*r* = −0.12)***, I (*r* = 0.12)***, sad, work, social (*r* = 0.12)***, see(*r* = 0.10)**, adverb (*r* = 0.10)***, percept (*r* = 0.10)**, apostrophe, ingest (*r* = 0.07)*, pronoun (*r* = 0.10)**, friend, time (*r* = 0.07)*, achieve	happiness	Age(*r* = −0.07)*	Self-description length, posts about 50–140 words/total posts	19
NFA avoidance	Religion, friend, death (*r* = 0.09)**, dash, exclusive, SpecArt, sexual, ingest, certain, period, Qmark, achieve	NA	NA	General emoticons-total words ratio(*r* = −0.06)*, bi-followers/followers, bi-followers/friends (*r* = 0.08)*	15

L, linguistic features; D, demographic features; B, behavioral features; NA means no features in this class were selected; *p < 0.05, **p < 0.01, ***p < 0.001(the Pearson correlation coefficient between selected features and questionnaire scores); Total, the total number of selected features for each dimension.

### 3.4. The performance of the models

We calculated correlations between the NFA total, NFA approach and NFA avoidance questionnaire scores and corresponding prediction scores output by the predictive models as a criterion validity index. To ensure the authenticity of the prediction results, training data couldn't be used as test data at the same time during model training.

When testing the models, the Pearson correlation coefficients between the actual questionnaire scores and the predicted scores of the test data were recorded during five-fold cross-validation. The mean of the five values of the correlation coefficients was calculated as “cross-validation result.” In addition, this cross-validation was performed five times to avoid potential biases due to data distribution changes after re-sampling. Finally, we obtained five “cross-validation results” and the mean of the five “cross-validation results” was calculated as the model validity index, namely the performance of the method (see Table 5). The results indicate that the performance of each algorithm varied, with XGB having the most optimal performance followed by GBR. Table 6 listed the five cross-validation results of XGB, indicating that the model validities of NFA total and two sub dimensions were fairly acceptable.

**Table 5:** The performance of models.

	**NFA total**		**NFA approach**		**NFA avoidance**	

	***r***	*R* ^2^	***r***	*R* ^2^	***r***	*R* ^2^
XGB	0.34**	0.11	0.31**	0.09	0.25*	0.04
LR	0.26*	0.04	0.21	0.02	0.2	0.03
SVR	0.09	0.001	0.12	0.009	0.08	0.002
LASSO	0.18	0.03	0.16	0.02	0.12	0.01
Ridge	0.17	0.02	0.13	0.01	0.12	0.01
ETR	0.27**	0.07	0.26*	0.06	0.22	0.05
RF	0.26*	0.06	0.26*	0.06	0.18	0.03
GBR	0.27**	0.06	0.27**	0.06	0.23	0.04

“*r*” is the Pearson correlation coefficient between predictive scores and NFA questionnaire scores (*p < 0.05, **p < 0.01); *R*^2^ is the proportion of the variability in the response variable; XGB, Extreme Gradient Boosting; LR, Linear Regression; SVR, Support Vector Regression; GBR, Gradient Boosting Regression; RF, Random Forest Regression; LASSO, Least absolute shrinkage and selection operator; Ridge, Ridge Regression; ETR, Extra Trees Regression.

**Table 6:** The five cross-validation results of XGB.

**Dimension**	**R_1_**	**R_2_**	**R_3_**	**R_4_**	**R_5_**	**M**	**SD**
NFA total	0.33	0.35	0.35	0.33	0.33	0.34**	0.01
NFA approach	0.3	0.33	0.3	0.31	0.31	0.31**	0.01
NFA avoidance	0.24	0.23	0.25	0.27	0.25	0.25*	0.01

*R*_1_–*R*_5_ are the results of the five times of 5-fold cross-validation; M is the mean of *R*_1_–*R*_5_ which indicates that the performance of XGB models for each NFA dimension (*p < 0.05, **p < 0.01); SD is the standard deviation of *R*_1_–*R*_5_.

### 3.5. The split-half reliability of the models

We also tested the split-half reliability of the prediction models. The posts of each participant in the test set were divided into two parts in chronological order, to extract linguistic features separately. The two sets of linguistic features were then combined with other features to form two sets of data namely “odd-data” and “even-data”. The test model was applied to these two sets to get the “split-half” scores. Pearson correlation coefficient between the two “split-half” scores was calculated as the split-half reliability index. As we can see in Table 7, the split-half reliability results of the two best-fitting models (XGB and GBR) achieved a high level of >0.60 on the NFA total, the NFA approach and the NFA avoidance, indicating the stability of the models. In addition, the models with the highest split-half reliability on NFA total and the other two dimensions were also the XGB models.

**Table 7:** The split-half reliability of models.

	**XGB**	**GBR**
NFA total	0.66***	0.62***
NFA approach	0.68***	0.66***
NFA avoidance	0.70***	0.70***

XGB, Extreme Gradient Boosting; GBR, Gradient Boosting Regression; ***p < 0.001.

## 4. Discussion

### 4.1. The feasibility of NFA prediction based on social media data

Based on public social media data, the present study has built prediction models using machine learning regression algorithms for identifying online users' NFA. Then the split-half reliability and criterion validity of the models were evaluated. Several models showed good reliability and validity, while the XGB algorithm performed best in both reliability and validity, and the GBR algorithm performed second. Both XGB and GBR are boosting algorithms, and it may have been suggested that boosting algorithms are more suitable for our datasets compared to other traditional machine learning algorithms. A possible explanation is that, in contrast to a single approach used in traditional algorithms, the iterative training of the base learner applied in the ensemble learning methods (XGB and GBR) provides a broader perspective on the data. Compared to GBR, XGB is an efficient and scalable variant of the gradient tree boosting (55, 56), and possesses the intrinsic ability to handle sparse features and situations where the class distribution is imbalanced (56–58). Some of the features in our datasets like “length of self-description” were sparse. Therefore, we presumed that's why XGB was more suitable for our datasets.

In our study, the model validity achieved the level of many previous studies on predicting other intrinsic psychological traits by social media data. For example, the Pearson correlation coefficients between the big-five personality scale scores and predicted scores were 0.29–0.40 (32). It indicates that identifying users' NFA based on social media data is also feasible.

### 4.2. The analysis of selected features

The selected features in our study also indicate some characteristics or behavior patterns of individuals with different NFAs. Our results demonstrate that people with different NFA levels tend to have different emotional expressions or experiences, which is consistent with previous studies (1, 12–14). From the selected features listed in Table 4, we have found that people with different NFA levels appear to have different emotional expression patterns in social media environments. The feature “general emoticons-total words ratio” refers to how many emoticons a person uses when expressing himself/herself. The ratio has positive correlation with NFA total (*r* = 0.10, *p* < 0.01) and negative correlation with NFA avoidance (*r* = −0.10, *p* = 0.05), indicating people with higher NFA are more likely to express their emotions online, that is consistent with previous studies (1, 2, 26). It also indicates that NFA implies general tendencies among various emotional experiences rather than engaging in specific affective experiences (13). The effective features of our predictive model also implies that the expression of emotions related to “happiness” and “sad” could reflect NFA approach, but the relationship between them seems not linear.

Our results also indicate that people with different levels of NFA may pay attention to different things in their online expressions. As we can see in Table 4, the feature “Death” is positively correlated with NFA avoidance (*r* = 0.10, *p* < 0.01) and negatively correlated with NFA total (*r* = −0.08, *p* < 0.01), which indicates people who tend to avoid emotion experiences are more likely to use death-related expressions in their posts. The result is consistent with previous research, which has shown that NFA avoidance is associated with a higher risk of suicide in nonclinical samples (3–5, 7–9). Studies on linguistic characteristics of suicidal people have comparably demonstrated that people with a high risk of suicide use death-associated expressions more frequently (e.g., “die” and “suicide”) (59, 60). In addition, religion-relevant expressions could reflect NFA avoidance. Differently, people with a higher NFA approach are more likely to use social-related expressions (e.g., “communication” and “discussion”) (*r* = 0.12, *p* < 0.001). This finding supports previous research on NFA: people with higher NFA are more likely to interact with others (1, 2, 26). Besides, work-related expressions could be associated with NFA approach.

Furthermore, other differences in online expressions and behaviors of people with different NFA levels have been observed. People with higher NFA approach prefer using pronouns (*r* = 0.10, *p* < 0.01) in online expression, particularly personal pronouns (*r* = 0.12, *p* < 0.001), where first-person singular use is significantly correlated with NFA approach (*r* = 0.12, *p* < 0.001). Prior research has pointed out that NFA approach potentially promotes life satisfaction (6), while people with high life satisfaction are more likely to express themselves in the first-person singular (61). Additionally, people who are high in need for affect are more likely to use perceptual related words (*r*= 0.10, *p* < 0.01), especially visual related ones (*r* = 0.10, *p* < 0.01). This finding is in line with previous research on individuals' preferences for information, that individuals with a high level of NFA preferred to process visual information (62).

Among demographic features, “Age” contributed to NFA approach model which had a negative correlation with the NFA approach (*r* = −0.10, *p* < 0.05). The same results were found in previous studies, the need for affect may decrease with age (1).

As Table 4 shows, among all the features involved in model training, SCLIWC had the most contribution to NFA prediction. This indicates that SCLIWC is a relatively effective tool to extract mental-related features from social media text, as some previous studies found in predicting psychological traits (42, 44). Of all the selected features, we can see the number of features that are significantly correlated with NFA avoidance is much less than those with NFA approach. It may explain why the NFA avoidance model had a lower criterion validity than the NFA approach.

### 4.3. The possible implications of NFA prediction

Present research proposed a measurement of NFA based on social media data, namely the NFA prediction model, and demonstrated its feasibility. This method does not require users to provide additional time costs to fill out questionnaires, which is an automatic, low-cost, and low-intrusive method to identify users' NFA.

NFA prediction has comprehensive applications. As individuals with different NFA prefer different types of services and products, e.g., movies (23) and product brands (19), understanding consumers' NFA is helpful for businesses to recommend and provide more user-friendly products. Based on the conclusion that NFA has a significant relationship with some mental health symptoms and suicidal tendencies (3–8), NFA prediction could help mental health workers provide more efficient and effective mental health services, including large-scale mental health monitoring and novel suicide interventions. With regard to public policy and public communication, the effectiveness of persuasive communication can be promoted via matching messages with the targeted audiences' affective orientation (14). Understanding differences in users' affective intrinsic motivation could help to tailor persuasive messages, facilitating the dissemination of positive and valuable information. In summary, NFA prediction can be used in a variety of scenarios requiring large-scale NFA measurements.

### 4.4. Limitation and future work

The findings of this study have to be seen in light of some limitations. First, like many other studies based on social media, the participants in this study were randomly recruited. Most of them were under the age of 30 (96%), which means the majority of participants were adolescents and young adults. To some extent, the results may be more applicable to adolescents. Additionally, although there was no gender difference in NFA total, NFA approach and NFA avoidance in our data, 78% of the participants were female, and this gender imbalance may bring some bias to our study. Second, although we have tried to eliminate some unqualified participants, the information provided by participants on social media may not always be correct and trustworthy. Third, for linguistic features, only dictionary-based word frequencies were extracted. There might be more attributes in the blog itself that may depict users' NFA, such as frequency domain features of time series data, and the mood or cognition changes between different blogs. However, these attributes have not been investigated. Fourth, in addition to the features used in this study, pictures and videos posted by users may also contain a lot of NFA-related information, which have not been used in model training. Fifth, increasing the sample size helps to improve the accuracy of the machine learning model, hence our model also requires more user data for analysis and accuracy improvement.

Despite these shortcomings, our results have implications for future studies on identifying NFA through social media. Further studies will continue to collect more social media data to train NFA models. Besides, they will explore more attributes such as pictures and videos to further improve the performance and reliability of the prediction model. Additionally, future studies could use a different set of features and compare the results across the features to put forward the most appropriate set of features related to NFA.

## Data availability statement

The datasets presented in this article are not readily available because the raw data cannot be made public. If necessary, we can provide feature data. Requests to access the datasets should be directed to the corresponding author NZ, zhaonan@psych.ac.cn.

## Ethics statement

The studies involving human participants were reviewed and approved by Scientific Research Ethics Committee of Institute of Psychology, Chinese Academy of Sciences (ethical code H15009). The patients/participants provided their written informed consent to participate in this study.

## Author contributions

HD performed the statistical analysis, trained the NFA models, and wrote the manuscript with input from all authors. NZ contributed to the conception and design of the study, the data collection, and the revision of the manuscript. YW helped to collected the data and revised the statistical analysis. All authors contributed to the article and approved the submitted version.
